# Eosinophilic esophagitis auxiliary diagnosis based on a peptide ligand to eosinophil cationic protein in esophageal mucus of pediatric patients

**DOI:** 10.1038/s41598-022-16293-1

**Published:** 2022-07-18

**Authors:** Tafarel Andrade de Souza, Ana Paula Carneiro, Andreia S. Narciso, Cristina P. Barros, Douglas Alexsander Alves, Luciane B. Marson, Tatiane Tunala, Tânia M. de Alcântara, Yara Cristina de Paiva Maia, Peter Briza, Fatima Ferreira, Luiz R. Goulart

**Affiliations:** 1 Laboratory of Nanobiotechnology Prof. Dr. Luiz Ricardo Goulart Filho, Institute of Biotechnology, Federal University of Uberlandia, Uberlandia, MG Brazil; 2 Pediatric Department, Federal University of Uberlandia, Uberlandia, MG Brazil; 3 Pathology Laboratory, Clinical Hospital, Federal University of Uberlandia, Uberlandia, MG Brazil; 4 Nutrition and Molecular Biology Research Goup, School of Medicine, Federal University of Uberlandia, Uberlandia, MG Brazil; 5 Department of Biosciences, University of Salzburg, Salzburg, Austria

**Keywords:** Biotechnology, Immunology, Biomarkers, Diseases, Gastroenterology, Medical research

## Abstract

Eosinophilic esophagitis (EoE) is a chronic inflammatory condition of the esophagus characterized by increased number of eosinophils. Currently, EoE diagnosis is based on endoscopic procedures for histopathological examination, eosinophils’ counting and, often, in clinical practice, the challenge is the differentiation between EoE and gastroesophageal reflux disease (GERD). Our aim was to develop novel peptide ligand to Eosinophil cationic protein (ECP) present in EoE biopsies of patients with potential to be used for detection. We performed a comparative proteomic analysis using liquid chromatography-tandem mass spectrometry (LC–MS/MS) of esophageal biopsies from pediatric patients with eosinophilic esophagitis, gastroesophageal reflux disease and control individuals. Then, phage display technology was used to select peptides against specific up-regulated protein from EoE patients. Twelve phage clones were selected after three biopanning rounds, and the best phage clone reactivity was evaluated by phage-ELISA assay using esophageal mucus samples from 94 pediatric patients. Mass spectrometry showed that eosinophil cationic protein (ECP) was one of the most up-regulated proteins in EoE patients, which is an eosinophil granule protein usually deposited on tissues to mediate remodeling, but in excess may cause fibrosis and hypertrophy, especially in allergic responses. A highly reactive ECP-ligand peptide (E5) was able to distinguish reactive mucus of EoE patients from GERD and the control individuals by Phage-ELISA, achieving a sensitivity of 84.62%, and a specificity of 82.72%. This is the first study that successfully demonstrated an antibody-like peptide targeting ECP at the esophagus mucus as a useful auxilliary tool for EoE diagnosis with a significant association with atopic disorders and dysphagia.

ClinicalTrials.gov no.: NCT03069573.

## Introduction

Eosinophilic esophagitis (EoE) is a chronic, allergic and inflammatory disease characterized by increased number of eosinophilic infiltrates in the esophageal mucosa, with severe hyperplasia in the squamous epithelium of the esophagus. The typical clinical symptoms of EoE are dysphagia, chest pain and food impaction resulting in esophageal dysfunction^1–4^ and fibrosis^5^.

Food antigens are the main precursors of the immune response of EoE patients^6^, although aeroallergens, such as pollen and fungi, may also be inducing factors ofesophageal eosinophilia^7,8^, but with limited evidence^3^.

Currently, the diagnostic criteria of EoE include esophageal dysfunction and eosinophilic infiltration, with any other associated inflammatory cause, especially gastroesophageal reflux disease (GERD)^9^. Endoscopic signs and histological changes are important for the characterization of EoE patients^9,10^. Among endoscopic signs, it is possible to observe fixed rings (trachealisation), transient esophageal rings (felinization), whitish exudate, longitudinal furrows, mucosal edema, esophageal narrowing and esophageal lacerations caused by endoscopy (crepe paper appearance). Histologically, EoE is determined by esophageal eosinophilia with presence of 15 or more eosinophils per high-power field (hpf)^2^. The international consensus was updated on diagnostic criteria for eosinophilic esophagitis (EoE)^11,12^ and in clinical practice, the challenge is the differentiation between EoE and GERD^13^, since these two diseases have considerable clinical and immunological overlaps^13–15^.

EoE treatment includes restriction of some food groups based on empirical or allergenic tests^15–17^, esophageal dilatation in the case of stenotic patients and use of (topical or systemic) corticosteroids^10,18^. The main problem of corticosteroid therapy is the clinicopathological remission of most patients after treatment discontinuation^18–20^. Evaluation of the efficacy of EoE treatments is confirmed by improvement of clinical signs and decrease of esophageal eosinophilia. However, it is necessary that patients undergo new endoscopic examinations to reevaluate biopsies^21^. Thus, investigations have been seeking disease biomarkers that may be useful for EoE diagnosis and prognosis.

Eosinophils and their granule proteins are involved in body defenses against helminth parasitic infections in inflammatory infiltrates of late-stage immune response, and may still be responsible for deregulated immunological reactions associated with allergic diseases, such as asthma, atopic dermatitis, EoE and other hypereosinophilic syndromes. Eosinophils may also play an important and constructive role in the maintenance of homeostasis, when involved in the body defense through immunological regulation, tissue repair and remodeling^22–24^.

Candidate biomarkers could be selected from the EoE pathogenesis, which involves Th2-mediated response to allergens^25–28^. A number of biomarkers of eosinophil activation, such as granule proteins^29–32^, have been shown to be elevated in EoE when compared to the controls, but none have been efficiently used.

Our aim in this investigation was to develop a ligand peptide selected by phage display against the eosinophil cationic protein (ECP), a highly expressed protein in patient’s biopsies, in order to distinguish EoE from others esophageal disease conditions. Due to current EoE diagnostic and management, we have used for the first time the patients’ mucus to validate a ligand peptide to ECP, which is highly secreted from affected tissues, a diagnostic platform that is discussed herein.

## Results

### Baseline characteristics of patients

Ninety-four subjects were eligible in the study and their baseline characteristics, such as clinical symptoms, allergies and summary of histological and gross features of esophageal mucosal are shown in Table 1. Eosinophil numbers were significantly greater in subjects with EoE compared with GERD and Control subjects. At the time of esophagogastroduodenoscopy (EGD) and sample colection, the mean patient age of EoE subjects was 8.3 years (± 4.8 years) with 69.2% being male, while in GERD subjects the mean patient age was 9.4 years (± 2.1 years) (53.8% male), and in Control subjects the mean patient age was 8.8 years (± 3.7 years) (48.5% male). EoE patients had ≥ 15 eos/hpf in proximal esophagus (33.9 ± 28.4) and distal esophagus (42.1 ± 35) at the time of analysis, while GERD subjetcs had < 15 eos/hpfin proximal esophagus (1.5 ± 1.8) and in distal esophagus (5 ± 4.9) and Control subjects had < 15 eos/hpf in proximal esophagus (0.8 ± 1.2) and in distal esophagus (1.3 ± 1.2). Across all groups, subjects reported the first symptom, such as, abdominal pain, nausea-vomiting, dysphagia, epigastric pain-heartburn, food impaction or others. EoE patientes most commonly reported either abdominal pain (38.4%) or nausea-vomiting (38.4%) while nausea-vomiting was most common in GERD subjects (53.8%). Control patients most commonly reported abdominal pain (47.0%). Endoscopic findings were reported, with edema being documented most frequently across all groups. All groups, subjects reported atopic disorders, such as, asthma, rhinitis, atopic dermatitis, food allergy and atopic parents. EoE and control subjects most commonly reported rhinitis, respectively, 53.8% and 42.6%, while atopic parents was most common in GERD subjects.

**Table 1:** Baseline characteristics of patients included in the study.

	EoE (n = 13)	GERD (n = 13)	Control (n = 68)
Age, mean (s.d.)	8.3 (4.8)	9.4 (2.1)	8.8 (3.7)
Male sex, no. (%)	9 (69.2)	7 (53.8)	33 (48.5)
**First symptom, no. (%)**			
Abdominal pain	5 (38.4)	3 (23)	32 (47.0)
Nausea-vomiting	5 (38.4)	7 (53.8)	20 (29.4)
Dysphagia	1 (7.6)	–	2 (2.9)
Epigastricpain-heartburn	–	2 (15.3)	2 (2.9)
Food impaction	–	–	2 (2.9)
Others	2 (15.3)	1 (7.6)	10 (14.7)
**Endoscopic findings, no. (%)**			
Fixedrings	2 (15.3)	–	–
Exudate	7 (53.8)	2 (15.3)	2 (2.9)
Furrows	7 (53.8)	1 (7.6)	1 (1.4)
Edema	11 (84.6)	7 (53.8)	18 (26.4)
Strictures	1 (7.6)	1 (7.6)	–
Transiente rings	1 (7.6)	2 (15.3)	1 (1.4)
**Atopic disorders, no. (%)**			
Asthma	3 (23)	1 (7.6)	7 (10.2)
Rhinitis	7 (53.8)	5 (38.4)	29 (42.6)
Atopic dermatitis	3 (23)	–	5 (7.3)
Foodallergy	6 (46.1)	1 (7.6)	7 (10.2)
Atopic parents	3 (23)	7 (53.8)	25 (36.7)
**Esophageal eosinophilia (Eos/hpf)**			
Proximal esophagus, mean (s.d.)	33.9 (28.4)	1.5 (1.8)	0.8 (1.2)
Distal esophagus, mean (s.d.)	42.1 (35)	5 (4.9)	1.3 (1.2)

*EoE* eosinophilic esophagitis, *GERD* gastroesophageal reflux disease, *Eos/hpf* eosinophils per high-power field.

### Mass spectrometry analysis

LC–MS/MS identified Eosinophil cationic protein (ECP) up-regulated in the EoE patients, with statistically significant differential expression between groups. Data of the three methods, MaxQuant/Perseus, PEAKS Studio (quantification with the built-in Q-Module) and PEAKS Studio (manual quantification and data validation after database search) were combined and visualized as bar graphs (mean + SEM) (Supplementary Fig. 1A). To compare Control, EoE and GERD groups, the average signal intensity ratio of all groups was calculated (Supplementary Fig. 1B). Identified peptide sequences of ECP and post-translational modification were analysed by mass spectrometry (Figure Supplementary 1C).

### Biopanning of ECP-ligand phages

**Figure 1:**
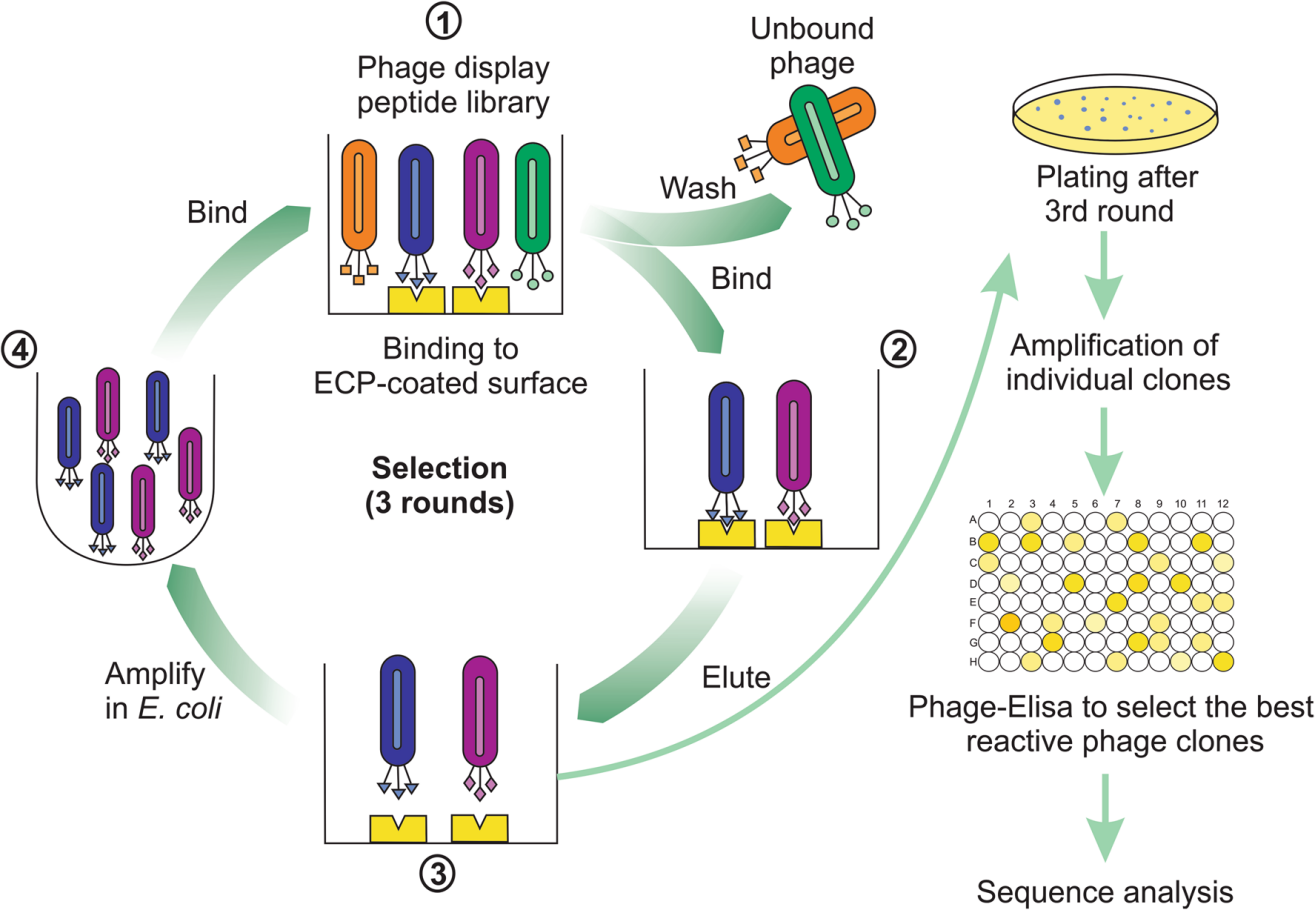
A scheme of the biopanning experiment. Phage library was incubated with Eosinophilic Cationic Protein (ECP) coated on surface. Unbound phages were washed and phages bound to ECP were eluted and amplified for next round of biopanning. After the 3rd round the eluate was plated and individual clones were amplified. Then, the most reactive phage clones were selected by Phage-ELISA for sequencing analysis.

**Figure 2:**
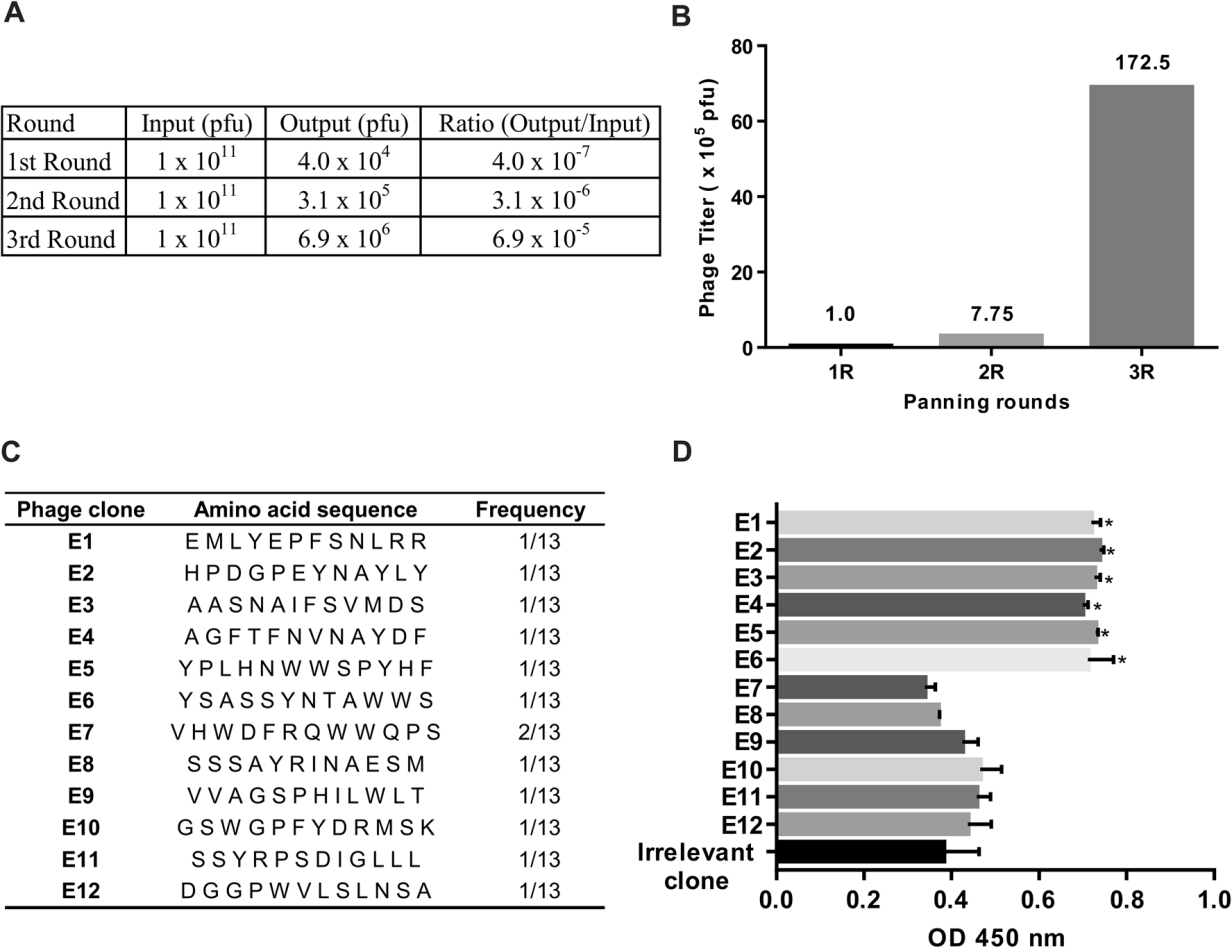
Biopanning, enrichment of phage library and perfomance of the phage clones selected by phage display. (**A**) The phage titer after each biopanning round, (**B**) Three rounds of biopanning against ECP were performed, and the phage titer (pfu) after each round was measured. Numbers on bars are the enrichment fold of the phage titer over the first round, (**C**) amino acid sequences and frequency of the selected peptides, (**D**) reactivity obtained though the interaction of the sequenced phage clones, Irrelevant phage clone and ECP.

Three rounds of biopanning were performed to screen M13 phage library against ECP (Fig. 1). The enrichment of phages was monitored by measuring titers of the output after each biopanning round and the fold enrichment relative to the titer of the first round. The phage titer was increased from the first round (4 × 10^4^ pfu) to the third round (6.9 × 10^6^ pfu) (Fig. 2A) and at the end of third round of biopanning, the phage titer was enriched to 172.5 folds over the first round (Fig. 2B). The enrichment of phage titer suggests that the biopanning of phages that selectively bind to ECP is successfully achieved.

A total of 96 phage clones were randomly picked after plating the third round. From 96 selected clones, 12 showed significant reactivity to ECP in the first screening by phage-ELISA, then these phage clones were subjected to polymerase chain reaction and DNA sequencing in order to translate the peptide sequences. After sequencing, the peptide sequences and their frequencies were analyzed (Fig. 2C). Reactivity values on phage-ELISA (1 × 10^10^ pfu/well) were very similar for the six different phages clones, which showed the highest absorbance values (Fig. 2D) and the highest difference compared to a irrelevant phage clone (1 × 10^10^ pfu/well) (*p* < 0.05).

### Validation of phage clones by phage-ELISA and ECP-E5 molecular docking

**Figure 3:**
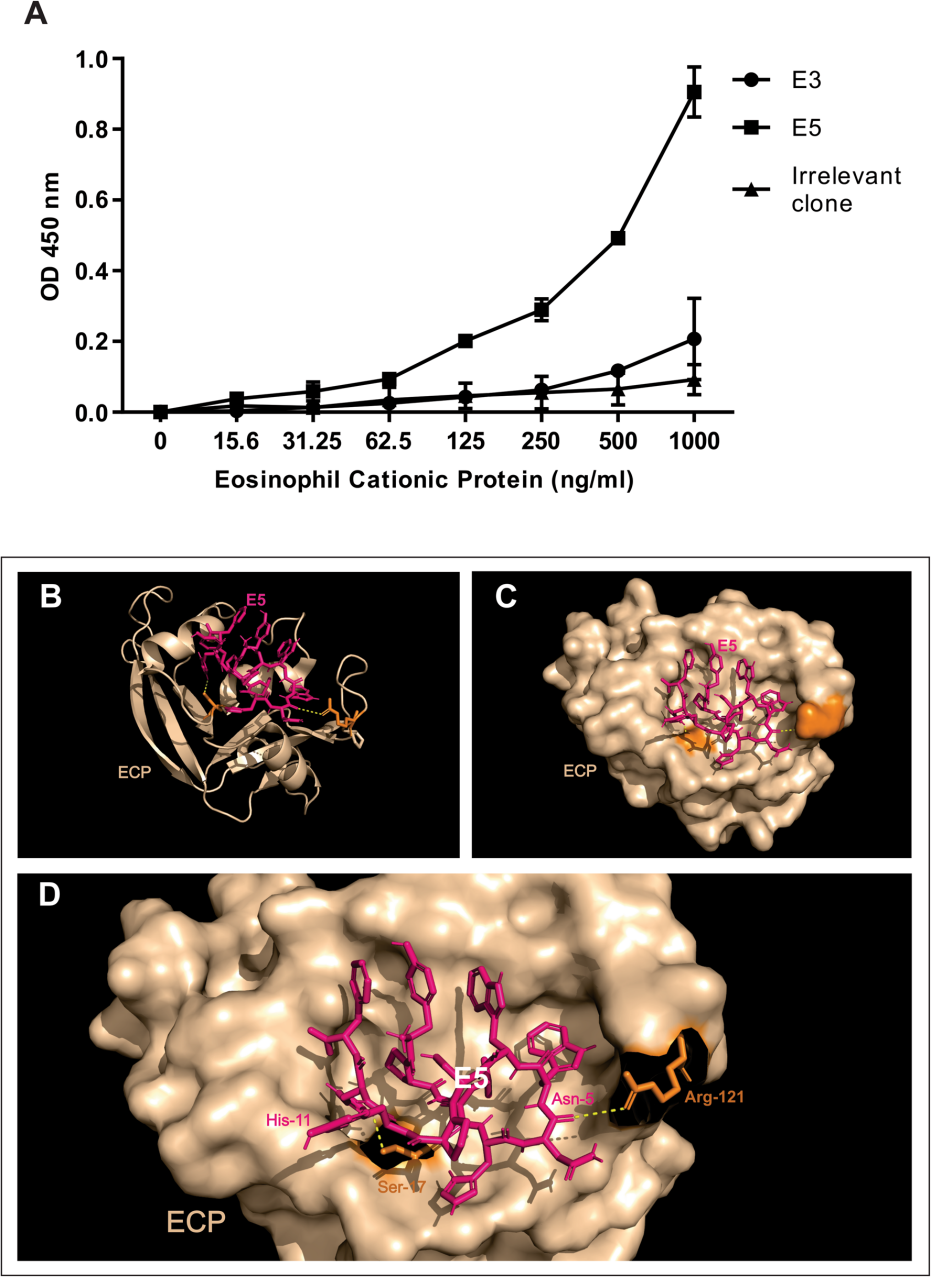
Detection of ECP using Phage-ELISA and molecular docking between the recombinant human Eosinophil Cationic Protein (ECP) and E5 peptide. (**A**) Increasing concentrations of ECP were pre-coated on plates and then incubated with the selected peptides E3, E5 and Irrelevant phage clone and then, incubated with anti-M13 antibody. Antibody was detected by HRP-conjugated anti-IgG and the enzyme substrate. Optical density (OD) was measured at a wavelength of 450 nm. Data represent mean OD ± standard deviation of assays performed in duplicates. (**B**) The full cartoon structure of ECP (wheat) coupled with E5 (hot pink), (**C**) top view of interaction surface topography revealing a binding pocket favoring the best affinity, (**D**) identification of E5 binding residues and interaction sites onto ECP. ECP binding residues are shown in orange. Yellow dashed lines represents polar contacts.

From the 12 clones sequenced, two clones with high absorbance values (using 1 × 10^10^ pfu/well) were tested to demonstrate their binding activity to ECP in a concentration-dependent manner using 1 × 10^9^ pfu/well. The E5 clone showed higher binding to ECP than the E3 clone, while the irrelevant phage clone showed weak binding to ECP. These results indicate the selective binding activity of these phage clones to ECP. The E3 clone was able to bind to ECP in a concentration-dependent manner, but the binding curve obtained by the E5 peptide was more linear and steeper according to the ECP concentrations (0–1000 ng/ml) (Fig. 3A). The successful modeling on E5 peptide and its most favorable interaction position with ECP protein was obtained, Fig. 3B shows the full cartoon structure of ECP (wheat) coupled with E5 peptide (hot pink). Figure 3C presents the surface structure of ECP forming a binding pocket which allows the peptide highest binding affinity energy (-5.8 kcal/mol). Figure 3D shows a zoomed view of the interaction site from docking analysis, where it is possible to see the polar contacts (yellow dashed lines) between the ECP’s Arg-121 and Ser-17, binding to E5’s Asn-5 and His-11 residues, respectively.

### Phage-ELISA of patients’ mucus

**Figure 4:**
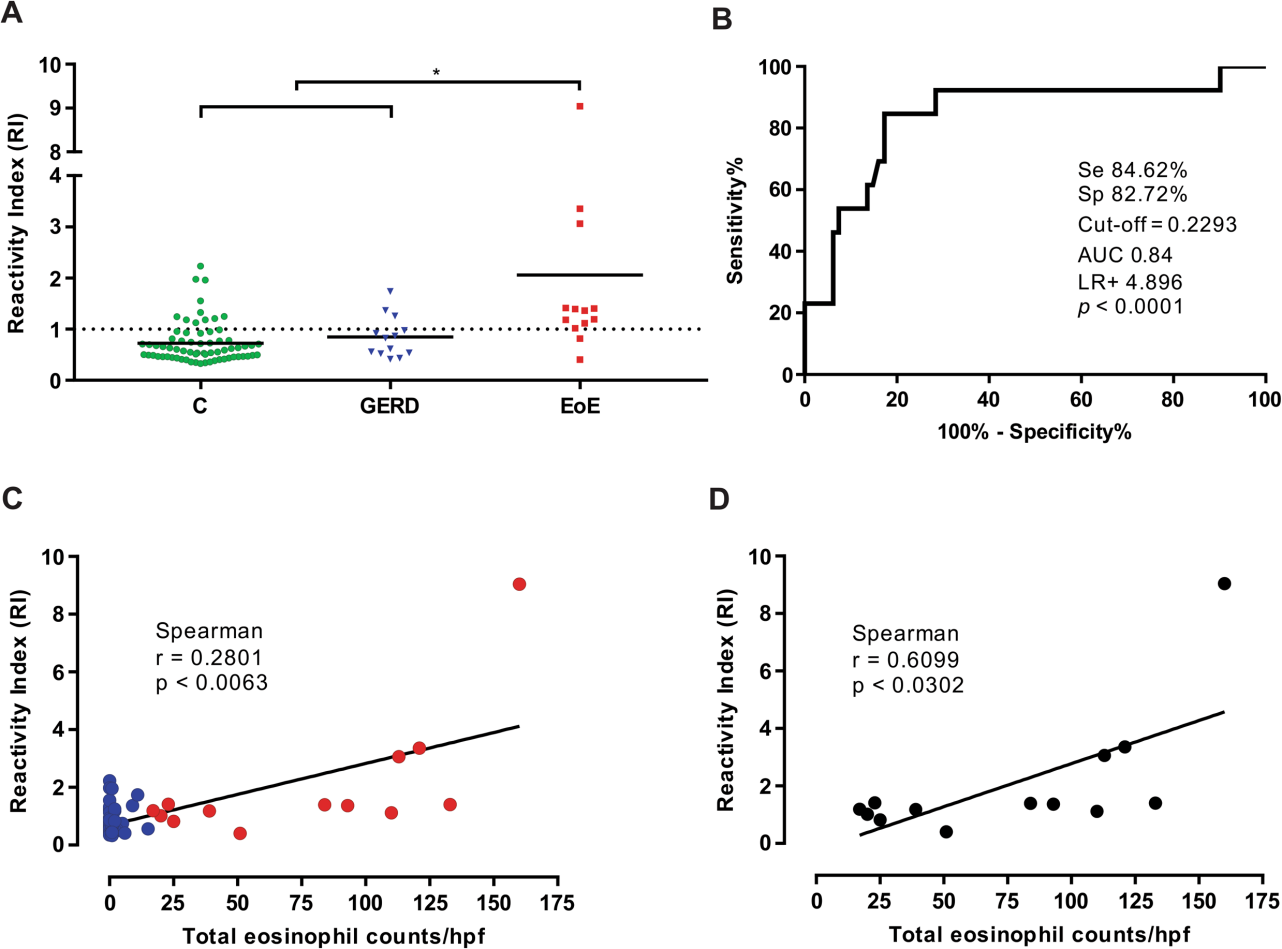
Phage-ELISA on mucus from patients. (**A**) Reactivity Index (RI) from the Phage-ELISA performed for the 94 patients classified in 3 groups: C (control), GERD (gastroesophageal reflux disease), and EoE (eosinophilic esophagitis). A diagnosis cut-off Phage-ELISA = 0.2993 at RI = 1 (datashed line) was derived from later, larger-scale studies by receiver operating characteristic (ROC) analysis, (**B**) ROC curve based on 0.2293 cut-off, with an area under the curve (AUC) of 0.84 and Se 84.62%, Sp 82.72%, LR + 4.896 and (*) p < 0.0001, (**C**) for the 94 patientes a linear correlation was performed between reactivity index (RI) and PEC (Eos/hpf). In blue circles (control and GERD) and in red circles (EoE), Spearman r and p value are shown in the figure, (**D**) For the 13 EoE patients a linear correlation was performed between reactivity index (RI) and PEC (Eos/hpf), Spearman r and p value are shown in the figure.

Phage-ELISA was performed to demonstrate whether the selected peptide could efficiently bind and detect ECP in the patients’mucus. The ECP specific peptide ligand (E5) was able to detect ECP in mucus samples. The Reactivity Index (RI) threshold was 0.2293, established by using receiver operating characteristic (ROC) curves (Fig. 4A). The ROC analysis demonstrated a good diagnostic value with area under curve (AUC) of 0.84, with sensitivity and specificity of 84.62% and 82.72%, respectively (Fig. 4B). A positive correlation was observed between the Reactivity Index (RI) and Peak Eosinophil Count/hpf (Fig. 4C, r = 0.2801, p < 0.0063), with a greater association between Reactivity Index (RI) and Peak Eosinophil Count/hpf within EoE patients (Fig. 4D, r = 0.6099, p < 0.0302). Patients with RI ≥ 1 pointed to a significant association between atopy and EoE, although some false positives in non-EoE patients have been observed that could be EoE patient. Interestingly, 64% of the 25 patients who had RI ≥ 1 presented allergic reactions. Rhinitis was found in 40% of patients RI ≥ 1. Atopic disorders in patients with RI ≥ 1 of each group (Supplementary Table 1) and the EoE characteristics present in non-EoE patients with RI ≥ 1 and in EoE patients with RI ≤ 1 are shown in Table 2.

**Table 2:** Features suggestive of EoE present in patients non-EoE with RI ≥ 1 and in EoE with RI ≤ 1.

Patient (group, no.)	RI Elisa	PEC (eos/hpf)	Gender	Dysphagia/food impactation	Atopic disorders	Histologic findings	EGD findings
C 07	1.241	0	F	Yes	AD, FA, AR	DIS	NL
C 13	1.184	0	M	No	–	NL	NL
C 15	1.206	0	M	No	AP	DIS	NL
C 17	1.324	0	M	Yes	FA	DIS	NL
C 23	1.976	0	F	No	–	DIS	NL
C 33	1.550	0	M	No	–	DIS	NL
C 52	1.123	0	M	No	–	DIS	NL
C 59	2.231	0	M	No	–	BLH, DIS	NL
CE 35	1.958	1	F	Yes	–	DIS	NL
CE 40	1.182	2	F	Yes	AR	DIS	NL
CE 68	1.249	2	M	Yes	AD	DIS	Edema
G 25	1.258	0	M	Yes	–	DIS	Exudate, Edema
GE 06	1.365	9	F	No	AR, AP	Eodegranulation	Furrows, exudate
GE 84	1.738	11	M	Yes	AR	BLH, DIS e eodegranulation	Stricture
E 19	0.816	25	M	Yes	AR	DIS	Furrows, edema
E 79	0.406	51	F	No	AD, FA	DIS	NL

*C* control without eosinophils, *CE* control with ≤ 15 eosinophils/hpf, *G* gastroesophageal reflux disease without eosinophils, *GE* gastroesophageal reflux disease with ≤ 15 eosinophils/hpf, *E* eosinophilic esophagitis, *AD* atopic dermatitis, *FA* food allergy, *AR* allergic rhinitis, *AP* atopic parents, *DIS* dilated intercellular spaces, *BLH* basal layer hyperplasia, *Eo* eosinophil, *EGD* esophagogastroduodenoscopy, *PEC* peak eosinophil counts, eosinophils/hpf, *NL* normal, *M* male, *F* female.

## Discussion

Currently, EoE diagnostic and therapy monitoring require multiple endoscopic procedures, overloading the health system, and justifying the urgent need of biomarkers to detect disease activity. Peripheral blood biomarkers have been associated with EoE, but none has been useful. Total IgE, eosinophil cationic protein (ECP), eosinophil-derived neurotoxin, tryptase, numerous cytokines, and a fraction of exhaled nitric oxide have been studied^31–33^. Only the number of peripheral blood eosinophils has proven to have correlation with the degree of esophageal eosinophilia, which was further evidenced by diminished levels after treatment, although their accuracy for diagnosis and their evaluation of disease activity are suboptimal^34^. Eosinophils contain unique cytoplasmic granules that degranulate under stimulation, releasing toxic mediators that can produce tissue damage and inflammation^25^, and their presence in esophageal tissue may be used as diagnostic tools to distinguish EoE patients^30,35,36^. Most of these methods require antibodies to detect eosinophil biomarkers. In this study, we have selected 12-mer peptides by phage display that strongly bind to ECP in patients’ mucus, demonstrating its clinical utility in the evaluation of EoE patients. The clinical performance of the phage-ELISA assay was validated using EoE and GERD patients, and controls. Our selection process led to a gradual enrichment of phage titers, and the best peptide candidate (E5), was the one with the highest affinity in the nanomolar range and selectivity to ECP.

We have demonstrated that the ECP-ligand peptide could efficiently detect ECP on mucus at a concentration as low as 25 ng/mL, and the EoE patients is the specifically population which benefits from testing the ECP-ligand peptide with sensitivity and specificity of 84.62% and 82.72%, respectively. Since endoscopic findings alone do not reliably establish the EoE diagnosis^37–39^, the selected peptide can also be used as an alternative to antibodies for ECP detection. Increased levels of ECP were significantly associated with atopy and EoE (100%). However, it is important to emphasize that false positives in non-EoE patients (64%) also presented atopies, suggesting that this marker may be indicative of allergic reactions that could lead to EoE. This hypothesis should be investigated. It is note worthy to mention that ECP can be elevated in other atopic diseases, such as asthma or allergic rhinitis^40^. It has been known that allergic rhinitis is significantly more common among EoE patients compared with healthy controls, but it remains unproven that atopy leads to EoE^41^. We have also found that 50% of patients in the EoE group presented food allergy, and IgE-mediated food allergies are common in EoE patients^42^.

Peak eosinophil count (PEC) of 15 or more in at least 1 hpf of esophageal biopsy remains the consensus threshold for histological diagnosis, but other pathological features have also been associated with active EoE. Recently, a study has described an EoE histological scoring system (EoEHSS) to objectively assess the severity and extent of multiple pathological features of EoE within esophageal biopsies. This score composed by the EoEHSS system exceeded the PEC in the differentiation of treated and untreated EoE patients^43^. In our study, non-EoE patients with RI > 1 (false–positive) had elements of the EoEHSS system, such as dilated intercellular spaces (DIS) and basal layer hyperplasia (BLH), which are histological elements that are associated with active EoE. This fact demonstrates that we are possibly identifying EoE patients in the non-EoE group, in which the standard diagnosis (eosinophil count) could not identify the disease in the biopsy. In this context, it is worth emphasizing that the dilation of the intercellular spaces can be a trigger mechanism that allows the penetration of the antigen in the epithelial barrier and for its presentation by esophageal dendritic cells^44^. In addition, most of these non-EoE patients have endoscopic features, atopic disorders and dysphagia that are characteristic of EoE^34^.

To the best of our knowledge, this is the first study that shows an antibody-like peptide that is capable of binding ECP in the mucus of EoE patients, with high sensitivity and specificity, substituting monoclonal antibodies that are difficult to stabilize. In addition, the use of mucus is highly desirable in EoE management and could be obtained through a minimally invasive device called an esophageal string test (EST), as demonstrated by Furuta et al.^45^, which is lower in cost compared to the current endoscopic mucosal sampling. Until now, references on ECP, as a serum biomarker used for monitoring patients on treatment with diets or pharmacological treatment with corticosteroids or PPIs, have shown consistent but insignificant reduction in ECP levels^20,35,46^. In this sense, we propose the use of this new peptide ligand to ECP in the mucus as a screening test of EoE that could complement the first endoscopy examination data to confirm diagnostic hypotheses and follow-up tool for EoE patients.

## Methods

### Patients and samples selection

This study was conducted between January 2015 and Septemper 2018 at the Clinics’Hospital of the Federal University of Uberlândia (HC/UFU) and all samples were analysed at the Laboratory of Nanobiotecnology (UFU). The study design was reviewed and approved by the Ethics and Research Committee of UFU under the protocol number CAAE 36787714.0.0000.5152. All methods were performed in accordance with the relevant guidelines and regulations. Written informed consent was obtained from all participants and/or their legal guardians. We have performed a prospective study of 94 children 1–16 years old from the HC-UFU Digestive Endoscopy Service undergoing esophagogastroduodenoscopy (EGD), and mucus samples were collected from distal to proximal esophagus using standard cytology brushes (Olympus BC-202D-5010) before obtaining esophageal biopsies. The brushes were dipped and stirred into the tube containing PBS. Four esophagus biopsies were taken from mid-proximal and distal levels. Patients were classified by consensus guidelines published in 2007 and updated in 2011^1,2^ by the new international consensus diagnostic criteria, recently published by Dellon et al.^11^ and summarized by Spergel et al^12^ (symptoms of esophageal dysfunction, ≥ 15 eosinophils per high power field [hpf]). The subjects enrolled in this study were classified into three groups: Patients with EoE, confirmed with pathology analysis of tissues from both distal and proximal esophagus with at least one biopsy fragment with ≥ 15 eosinophils/hpf (E; n = 13), patients with GERD, confirmed with the presence of any symptoms related to reflux disease considering the age of the patient, associated with erosion esophagitis or abnormal esophageal pH monitoring study for infants and children under the age of 8 and for older children and adolescents, the same criteria were used, adding heartburn improvement upon proton pump inhibitor (PPI) therapy (G; n = 13), and Control, consisted of patients whose esophageal epithelium was unremarkable, without esophageal eosinophilia, and their outcome did not reveal eosinophilic disorders or GERD (C; n = 68). A flow chart with endoscopic diagnostic procedures, clinical follow-up and laboratorial procedures was developed and applied in this study (Supplementary Fig. 2). Exclusion criteria were receiving acid suppressed therapy and corticoids in the last 4 weeks, congenital or acquired esophageal stenosis, and previous diagnoses related to eosinophilia at the moment of the samples’ collection. Histories of various clinical symptoms, allergies, endoscopic and pathological studies were collected.

### Mass spectrometry

Proteins from esophageal biopsies were identified by mass spectrometry. Using label-free quantification, protein levels were compared between samples from pediatric patients. Well-defined patients from the control (C; 7), EoE (E; 3) and GERD (G; 3) groups were compared. The extraction step started with digestion of 10 mg/sample using the ProteoExtract^®^ All-in-One Trypsin Digestion Kit (EMD Millipore, Billerica, MA, USA) as described elsewhere with a few modifications^47^. Aproximately 10 mg of tissue were added to 200 µL extraction buffer 2 and 200 µL of glass beads (0.5 mm), shaken for 1 h at 4 °C. Proteins were reduced, alkylated and digested in standard conditions for 3 h. Resulting peptides were desalted using C18 ZipTip (EMD Millipore) and separated by reverse-phase nano-HPLC (Dionex Ultimate 3000, Thermo Fisher Scientific, Bremen, Germany), column: PepSwift Monolithic Nano Column, 100 µm × 25 cm (Dionex). The column was eluted with an acetonitrile gradient (Solvent A: 0.1% (v/v) FA/0.01% (v/v) TFA/5% (v/v) DMSO; solvent B: 0.1% (v/v) FA/0.01% (v/v) TFA/90% (v/v) ACN/5% (v/v) DMSO; 5–45% B in 60 min) at a flow rate of 0.8 µL/min at 55 °C. Peptides were analyzed with a Q Exactive Orbitrap mass spectrometer (Thermo Fisher Scientific, Bremen, Germany) directly coupled to the HPLC. Capillary voltage at the nano electrospray head was 2 kV, the instrument was tuned for maximum sensitivity. Peptide fragmentation/identification was done with a top 12 method and a normalized fragmentation energy at 27%. Aliquotes of the extracts were analyzed five times. Consequently, five independent MS experiments per biopsy were done. Data of these five MS experiments were combined and analyzed with MaxQuant^48^and PEAKS Studio 8.5 (BSI, Waterloo, Canada), followed by visual data inspection and validation. Protein identification was performed at the Human UniProt Complete Proteome database.

### Materials

Ph.D-12mer phage peptide library was purchased from New England Biolabs (New England Biolabs, Beverly, MA, USA). This contains a structurally constrained 12-mer random peptide library with complexity of 1.2 × 10^9^ and *E. coli* ER 2738 as a host cell. Recombinant Human Eosinophil Cationic protein was purchased from Abcam (Cambridge, Massachusetts, USA). ELISA microplate 96-well, hingh binding PS, F-bottom, (Chimney Well), clear, Microlon®, were purchased from Greiner Bio one (Kremsmunster, Austria), bovine serum albumin (BSA) was from Bovogen (East Keilor, Australia), horseradish peroxidase (HRP)-conjugated anti-M13 antibody was from GE Healthcare (New Jersey, USA), 3,3’,5,5’-tetramethylbenzidine (TMB) substrate was from BD OptEIA™ (California, USA) and 5-Bromo-4-Chloro-3-Indolyl β-D-Galactopyranoside (X-Gal) was from Ludwig Biotecnology (Rio Grande do Sul, Brazil). Absorbance was measured using the Sunrise Basic microplate reader (Tecan group Ltd, Männedorf, Switzerland).

### Biopanning

ELISA microplate was coated with 100 µL of ECP (1 µg/well) in phosphate-buffered saline (PBS) at 4 °C overnight, then the plate was blocked for 1 h at 37 °C using BSA blocking buffer, 5% in PBS. Each biopanning round consists of selection of phages that binds to ECP and amplification of the eluted phages. In the first round, 10 µL of 1 × 10^11^ M13 phage library in 90 µL of PBS was added to well and incubated for 1 h at 37 °C with gentle shaking for subtracting the phages that binds to the ECP. After incubation, the unbound phages were extensively washed using PBS, with 0.05% Tween-20 (PBST 0.05%) to minimize the non-specific binding of phages. The bound phages were eluted by incubating with 150 µL of 0.2 M glycine (pH 2.2) at RT for 10 min and immediately neutralized with 22.5 µL of 1 M Tris- HCl (pH 9.1). The phage clones (100 µL) were amplified using ER2738 *E. coli* and precipitated using 20% polyethyleneglycol (PEG)/2.5 M NaCl and then suspended in PBS and used for next round of screening. The phage titration was performed by serially diluting the eluate and plated on Luria–Bertani (LB) media containing IPTG (200 mg/mL) and X-Gal (20 mg/mL) to visualize the colonies ER2738 *E. coli* infected by phage clones in blue as described elsewhere^49^. The same amount of input (1 × 10^11^ pfu of phages) was maintained in the subsequent rounds (Fig. 1).

### Phage binding ELISA assays

ELISA plates were coated with 100 µL/well of ECP (1 µg/ml) and BSA (1 µg/ml) as a negative control in 0.1 M carbonate/bicarbonate buffer (NaHCO_3,_ pH 9.6) at 4 °C overnight to select the best reactive phage clones. After washing two times with PBST 0.05%, the wells were blocked for nonspecific sites with 300 µL of BSA blocking buffer, 5% in PBS, incubating for 1 h at 37 °C. After washing the wells three times with PBST 0.05%, 100 µL of selected phage clones (1 × 10^10^ pfu/well) were added in BSA blocking buffer, 5% in PBS and incubated at 37 °C for 1 h, and was used irrelevant phage clone as a negative control. After washing the wells six times with PBST 0.05%,was added 100 µL per well of 1:5000 HRP–anti-M13 conjugate diluted in BSA blocking buffer, 5% in PBS and incubated at 37 °C for 1 h to detect the phage clones binding to ECP. Then the wells were washed six times with PBST 0.05% and 100 µL of TMB substrate was added and incubated at RT for 5–15 min. Finally, the reaction was stopped using 50 µL of 2 M H_2_SO_4_ and plates were read at 450 nm using amicroplate reader (Fig. 1).

### DNA extraction and sequencing

After 3^rd^ round of biopanning, the phage DNA of the best reactive phage clones selected by phage-ELISA were isolated from 1 mL overnight cultures by precipitation with 1/6 volume PEG/NaI (20% w/w, polyethylene glycol 8000) and iodide buffer (10 mM Tris–HCl (pH 8.0), 1 mM EDTA, and 4 MNaI). Phage DNA was precipitated with absolute ethanol, followed by a wash with 70% ethanol, and resuspended in 20 μl Milli-Q water as described elsewhere^49^. Electrophoresis was performed in 0.8% agarose gel stained with gel red solution in order to verify DNA quality. The inserts were confirmed by polymerase chain reaction of the phage DNA. DNA inserts of twelve individual clones were sequenced with the primer—96 M13 (5′-OH CCC TCA TAG TTA GCG TAA CG-3′ following the manufacturer’s instructions, and detection was performed in GenomeLab™ GeXP Genetic Analysis System (Beckman Coulter®) (Fig. 1).

### Bioinformatic analysis

The Recombinant Human Eosinophil Cationic Protein pdb was obtained from Protein Data Bank (PDB id: 1QMT)^50^. The E5 peptide (YPLHNWWSPYHF) tridimensional structure was modeled by using the PEP-FOLD3 software^51^. The best model was chosen based on software’s internal scores and online verification tools, such as SAVES v6.0^52,53^ RAMPAGE: Ramachandram Plot Assessment^54^, that together evaluates the stereochemistry and spatial coherence, of predicted molecule. After that, molecular docking was performed to predict the interaction of both structures. AutoDOCK Vina^55^ was used to simulate and predict the ECP-E5 interaction using the Root-mean-square deviation of atomic positions (RMSd) and free energy calculations. PyMOL Molecular Graphics System, Version 2.0 Schrödinger, LLC, was used for visualization, editing and exporting image files.

### Phage-ELISA of patients’ mucus

For phage-ELISA using mucus from patients, ELISA plates were coated with 4 µg/well of the soluble fraction protein of the esophageal mucus from each of the 94 patients in duplicate diluted in 0.1 M NaHCO_3_ pH 9.6, incubating overnight at 4 °C. After washing two times with PBST0.05%, the plates were blocked for nonspecific sites with 300 µL of BSA blocking buffer, 5% in PBS, incubating for 1 h at 37 °C. Then the wells were washed three times with PBST 0.05% and incubated with 100 µl/well of E5 phage clone diluted 1 × 10^9^pfu/well in BSA blocking buffer, 5% in PBS. After incubation, for 1 h at 37 °C the plates were washed three times with PBST 0.05%, and was added 100 µL per well of 1:5000 HRP–anti-M13 conjugate diluted in BSA blocking buffer, 5% in PBS. After 1 h at 37 °C the plates were washed six times with PBST 0.05%, and 100 µl of TMB substrate was added and incubated at RT for 5–15 min. Finally, the reaction was stopped using 2 M H_2_SO_4_ and plates were read at 450 nm using a microplate reader as described elsewhere^49^. All samples were tested in duplicate. The optimum point of reaction for anti-M13 antibody detection was determined using the receiver operating characteristic (ROC) curve, where a cut-off point was determined as the value of the parameter corresponding to the highest possible sensitivity without losing specificity. To calculate the ROC curve, sensitivity and specificity, we considered the Control and GERD groups as a single group (non-EoE). Each serum sample was tested without phage as negative control. The final OD values obtained for each mucus samples were adjusted by subtracting the corresponding OD value obtained by the negative control. After data adjustment, OD values obtained for each sample from all groups were divided by the cut-off value for data normalization. The values obtained are expressed as reactivity index (RI), where mucus samples presenting RI ≥ 1 were considered positives. The Reactivity Index (RI) of samples was calculated using the equation RI = sample absorbance/cut-off.

### Statistical analysis

Non-parametric Friedman's one-way ANOVA test analysis was used to evaluate the differences in phages clones ractivity to ECP in aconcentration-dependent manner in phage-ELISA. Non-parametric Mann Whitney test analysis was used to evaluate the differences in mucus samples’ reactivity in phage-ELISA assays among groups for E5 phage clone. Sensitivity and specificity parameters were calculated based on ROC curve analysis. To estimate the positive predictive accuracy, the area under the curve (AUC) was also determined. Spearman’s correlation was used for analysis among variables. Statistical analyses were performed using GraphPad Prism 7 software (GraphPad Software, Inc. San Diego, CA). *p* values less than 0.05 were considered statistically significant.

## Supplementary Information

Supplementary Information.
