# Supplementary material for: Eosinophilic esophagitis auxiliary diagnosis based on a peptide ligand to eosinophil cationic protein in esophageal mucus of pediatric patients
Source: Sci Rep. 2022 Jul 18;12:12226. doi: 10.1038/s41598-022-16293-1 (PMC9289663; doi:10.1038/s41598-022-16293-1)
Supplement: Supplementary file 1 — Supplementary Information. [file 41598_2022_16293_MOESM1_ESM.pdf]

TITLE: Eosinophilic esophagitis auxiliary diagnosis based on a peptide ligand to eosinophil cationic protein in esophageal mucus of pediatric patients

Tafarel Andrade de Souza[1,+,*], Ana Paula Carneiro[1,+], Andreia S. Narciso[1], Cristina P. Barros[2], Douglas Alexsander Alves[1], Luciane B. Marson[2], Tatiane Tunala[3], Tânia M. de Alcântara[3], Yara Cristina de Paiva Maia[1], Peter Briza[4], Fatima Ferreira[4], Luiz R. Goulart[1]

[1]Laboratory of Nanobiotechnology, Institute of Biotechnology, Federal University of Uberlandia, Uberlandia, MG, Brazil;
[2]Pediatric Department, Federal University of Uberlandia, Uberlandia, MG, Brazil;
[3]Pathology Laboratory, Clinical Hospital, Federal University of Uberlandia, Uberlandia, MG, Brazil;
[4]Department of Biosciences, University of Salzburg, Austria, Europe.

[+]these authors contributed equally to this work
***tafarelandradesouza@hotmail.com**

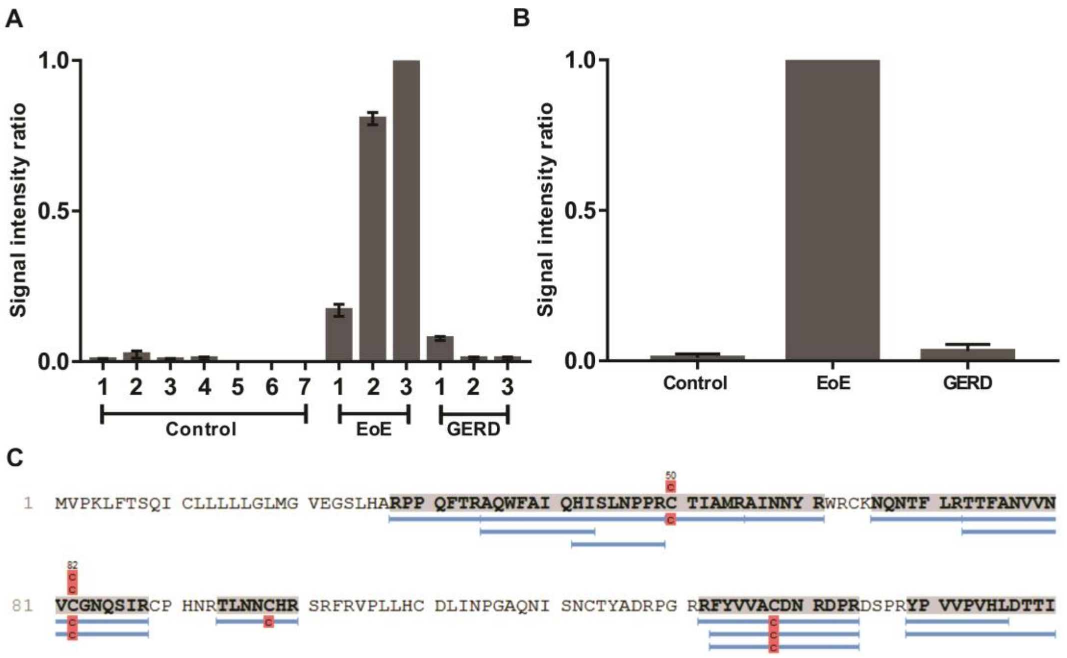

Supplementary Figure 1. Mass spectrometry analysis. (A) Representative samples of each group were analyzed - Control (1-7), EoE (1-3) and GERD (1-3). Data of the three methods for quantification of Eosinophilic Cationic Protein (ECP) were combined and visualized as bar graphs (mean±SEM), (B) To compare Control, EoE and GERD samples, the average of the signal intensity ratio from each type of samples was calculated (mean±SEM), (C) Natural ECP identified and analysed by mass spectrometry. Regions covered by identified peptides are shown in gray, each blue bar indicates an identified peptide sequence and the post-translational modification (PTM) carbamidomethylation is shown in red boxes.

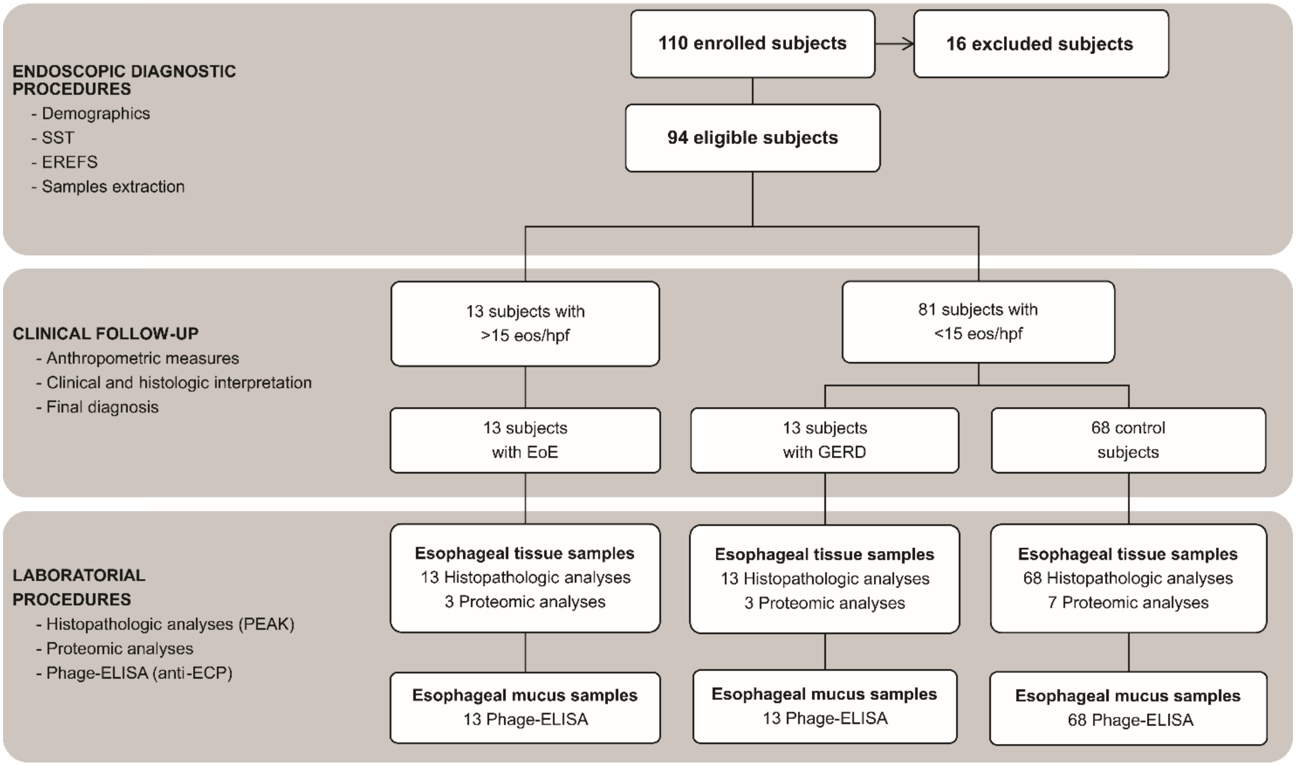

Supplementary Figure 2. The flow-chart of the study with the description of procedures and analysis . SST, symptom scoring tool; EREFS, endoscopic reference score; PEC, Peak eosinophil count, ECP, eosinophil cationic protein.

Supplementary Table 1. Atopic disorders in patients with RI ≥ 1.

	RI ≥ 1 (nº)	AnyAtopic disorders, nº (%)	Asthma, nº (%)	Rhinitis, nº (%)	Atopic dermatitis, nº (%)	Food allergy, nº (%)	Atopic parents, nº (%)
EoE	11	9 (81.8)	3 (27.2)	6 (54.5)	2 (18.1)	5 (45.4)	2 (18.1)
GERD	3	2 (66.6)	—	2 (66.6)	—	—	1 (33.3)
Control	11	5 (37.5)	—	2 (18.1)	2 (18.1)	2 (18.1)	1 (9)
Total	25	16 (64)	3 (12)	10 (40)	4 (16)	7 (28)	4 (16)

RI, Reactivity Index on ELISA; EoE, Eosinophilic Esophagitis; GERD, Gastroesophageal reflux disease; Eo, eosinophil.
